# *Asparagus* Spears as a Model to Study Heteroxylan Biosynthesis during Secondary Wall Development

**DOI:** 10.1371/journal.pone.0123878

**Published:** 2015-04-20

**Authors:** Lili Song, Wei Zeng, Aimin Wu, Kelsey Picard, Edwin R. Lampugnani, Roshan Cheetamun, Cherie Beahan, Andrew Cassin, Andrew Lonsdale, Monika S. Doblin, Antony Bacic

**Affiliations:** 1 Nurturing Station for the State Key Laboratory of Subtropical Silviculture, Zhejiang A & F University, Lin’an, Hangzhou, 311300, P. R. China; 2 ARC Centre of Excellence in Plant Cell Walls, School of Botany, the University of Melbourne, Parkville, VIC 3010, Australia; 3 College of Forestry, South China Agricultural University, Guangzhou, 510642, China; 4 Bio21 Molecular Science and Biotechnology Institute, the University of Melbourne, Parkville, VIC 3010, Australia; Iowa State University, UNITED STATES

## Abstract

Garden asparagus (*Asparagus officinalis* L.) is a commercially important crop species utilized for its excellent source of vitamins, minerals and dietary fiber. However, after harvest the tissue hardens and its quality rapidly deteriorates because spear cell walls become rigidified due to lignification and substantial increases in heteroxylan content. This latter observation prompted us to investigate the *in vitro* xylan xylosyltransferase (XylT) activity in asparagus. The current model system for studying heteroxylan biosynthesis, *Arabidopsis*, whilst a powerful genetic system, displays relatively low xylan XylT activity in *in vitro* microsomal preparations compared with garden asparagus therefore hampering our ability to study the molecular mechanism(s) of heteroxylan assembly. Here, we analyzed physiological and biochemical changes of garden asparagus spears stored at 4 °C after harvest and detected a high level of xylan XylT activity that accounts for this increased heteroxylan. The xylan XylT catalytic activity is at least thirteen-fold higher than that reported for previously published species, including *Arabidopsis* and grasses. A biochemical assay was optimized and up to seven successive Xyl residues were incorporated to extend the xylotetraose (Xyl_4_) acceptor backbone. To further elucidate the xylan biosynthesis mechanism, we used RNA-seq to generate an *Asparagus* reference transcriptome and identified five putative xylan biosynthetic genes (*AoIRX9*, *AoIRX9-L*, *AoIRX10*, *AoIRX14_A*, *AoIRX14_B*) with *AoIRX9* having an expression profile that is distinct from the other genes. We propose that *Asparagus* provides an ideal biochemical system to investigate the biochemical aspects of heteroxylan biosynthesis and also offers the additional benefit of being able to study the lignification process during plant stem maturation.

## Introduction

Heteroxylans represent a major family of non-cellulosic polysaccharides in dicot secondary walls and monocot primary walls [1]. They are composed of a linear backbone of 1,4-linked β-D-xylose (Xyl) residues substituted with variable side branches that are mostly composed of α-D-glucuronic acid (GlcA), 4-O-methyl-α-D-glucuronic acid (MeGlcA) and/or α-L-arabinofuranose (Ara*f*) depending on the species and tissue types [2]. Thus, heteroxylans are classified according to their backbone substitution patterns as either (methyl)glucuronoxylans ((Me)GXs), glucurono(arabino)xylans (GAXs) or arabinoxylans (AXs). (Me)GXs predominate in the secondary walls of dicot wood and non-commelinoid monocots, the grouping that includes garden asparagus (*Asparagus officinalis* L.) and many other economically valuable species. However, in the commelinoid monocots, represented by the commercially important cereals (grasses) such as rice, maize, wheat and barley, GAXs are the most abundant non-cellulosic matrix polysaccharides [3]. AXs are particularly rich in cereal endosperm walls, constituting up to 70% (w/w) of the endosperm cell wall in wheat [4].

Heteroxylans are synthesized in the Golgi apparatus (GA) and then transported via post-Golgi secretory vesicles to the plasma membrane where they are deposited into the wall [5]. The biosynthesis requires multiple type II glycosyltransferases (GTs) including xylosyltransferases (XylTs) responsible for generating the β-(1,4)-Xyl backbone and glucuronosyltransferases (GlcATs) and arabinosyltransferases (AraTs) responsible for incorporation of the major side chain residues [6]. In *Arabidopsis*, several GTs involved in heteroxylan biosynthesis have been identified via mutant analysis. These include members of the CAZy GT family 47 (*IRX10* and *FRA8/IRX7*) [7–9], GT43 (*IRX9* and *IRX14*) [10,11] and GT8 (*IRX8*, *PARVUS* and *GUX*) [12–14]. Among these GTs, IRX9, IRX10, and IRX 14 as well as their homologs (IRX9-Like (L), IRX10-L and IRX14-L) are believed to be essential for the elongation of the xylan backbone [8,10–12,15–17]. Additionally, several gene families responsible for the glycosyl residue substitution by non-glycosyl residues have been reported and include DUF579 for transfer of a methyl group to GlcA on the xylan backbone [18], BAHD involved in ferulic and *p-*coumaric acid addition [19] and TBL29 catalyzing Xyl acetylation [20,21].

In parallel, *in vitro* xylan biochemical assays have been developed in order to understand the molecular mechanism of their assembly. Feingold [22] first demonstrated that *Asparagus* microsomes could transfer D-Xyl to a β-(1,4)-linked xylo-oligosaccharide acceptor but only a single Xyl was reported to be incorporated. Subsequently, processive xylan-XylT activity has been confirmed in various species including maize [23], sycamore and poplar [24], pea [25], *Zinnia* [26], wheat [27] and *Arabidopsis* [10,15]. *Arabidopsis* GT43 mutants (*irx9* and *irx14*) have reduced xylan and xylan XylT activity, providing evidence that they play a role in xylan biosynthesis [15]. Tobacco microsomes from suspension-cultured cells co-expressing both *IRX9* and *IRX14*, but not *IRX9* and *IRX14* seperately, have been shown to have XylT activity, indicating that these two proteins act cooperatively and may be part of an active xylan biosynthetic complex [28]. A partially purified xylan biosynthetic complex was isolated from wheat seedlings and found to contain GT43, GT47 and GT75 proteins, suggesting their involvement in GAX biosynthesis [29]. Recently, two groups have independently demonstrated that heterologously expressed IRX10 has processive xylan XylT activity. Urbanowicz et al [21] expressed the *Arabidopsis IRX10-L* in mammalian HEK293 cells and Jensen et al [30] expressed separately a *Plantago ovata* and the moss (*Physcomitrella patens*) *IRX10* gene in *Pichia pastoris* and found that the heterologously expressed IRX10 proteins could add multiple Xyl residues onto a xylo-oligosaccharide acceptor. The *P*. *patens* homolog was described as displaying “robust” xylan XylT activity with the *Arabidopsis* and *P*. *ovata* homologs showing “lower but reproducible” activity [30]. However, it remains unclear whether the heteroxylan biosynthetic mechanism(s) between dicots and grasses are the same since dicot (and gymnosperm) xylans possess a distinct ‘primer’ oligosaccharide at the reducing end [31] unlike grass xylans that lack this specific sequence [32,33].

The monocot order Asparagales includes many economically important species, some with edible plant parts such as onion, garlic, asparagus and vanilla as well as many cut flower species including orchids, daffodils and irises. The ‘developmentally immature, rapidly growing’ subterraneous shoot (also referred to as the spear) of garden asparagus (*Asparagus officinalis* L.) is an important agricultural crop consumed for both its dietary fibre values and distinctive flavor. Post-harvest *Asparagus* ‘quality’, including its texture and flavor, deteriorates relatively quickly and this has been attributed to tissue maturation-related hardening [34]. This hardening is due to secondary wall formation and increased lignification of the secondary walls of the vasculature and supporting structures. This maturation process is also associated with a significant increase in heteroxylans [35] and xylan-pectic polysaccharide complexes [36], suggesting there is active xylan synthesis occurring in the *Asparagus* spear post-harvest.

In this study, we verified the changes in the polysaccharide composition and lignification process in *Asparagus* spears. We then biochemically characterized a surprisingly active xylan XylT activity in different stem sections (apical, middle and basal) over post-harvest stages. We found that the highest XylT activity is in the basal section of *Asparagus* spears, a region that also has high heteroxylan and lignin content. In addition, we generated a reference transcriptome to examine the expression of *Asparagus* XylT genes throughout the sections and storage stages. Together our results show that *Asparagus* is an ideal non-commelinoid monocot model system to study heteroxylan biosynthesis, particularly as it offers a tractable system to purify the *in vivo* protein complex responsible for the biochemical activity that will be essential in elucidating our understanding of the molecular mechanism of xylan synthesis.

## Materials and Methods

### Plant material

Green garden asparagus (*Asparagus officinalis* L.) spears were harvested from a local producer (Vizzarri Farm, KooWee Rup, Victoria,Australia) and transported on ice to the laboratory within 2 hr post-harvest. After gentle washing with distilled water, dry spears with a mean butt (bottom of the spear) diameter of 1.0–1.5 cm were cut to approximately 21 cm in length and marked at 7-cm intervals. The spears were subsequently sorted into bunches weighing approximately 600 g (~15 spears per bunch). Each bunch was placed into plastic containers and stored at 4±1°C for up to 16 days with ~95% relative humidity in the dark. *Asparagus* spears at harvest (0 day) and after 4, 8, 12 and 16 days of storage were cut into three 7-cm sections (apical, middle and basal) and the FW measured. For RNA extraction, the spears were frozen in liquid nitrogen and stored at -80°C prior to processing.

### Fresh weight (FW) loss

Weight loss was recorded periodically by weighting 15 spears during storage using a digital 3 decimal place balance (Mettler-Toledo, Melbourne, Australia). The results were expressed as the percentage loss of initial FW.

### Visual evaluation of *Asparagus* spear appearance

The general appearance of the spears was independently assessed by at least 10 trained panelists and scored using a 5 to 1 scale, where 5 = excellent, 4 = very good, 3 = good, 2 = fairly good, 1 = bad. The appearance index was calculated as Σ = appearance scale × percentage of corresponding spears within each class. A minimum value of 3 is required to be considered as commercially acceptable [37].

### Determination of spears texture

*Asparagus* spear texture was determined using a TA-XT2i texture analyzer (Stable Micro Systems, England) fitted with a 5 mm diameter probe. The penetration rate was 1 mm/s with a final penetration depth of 10 mm. The maximum of weight was recorded through the curve. Data were expressed as kg cm^-2^.

### Cell wall polysaccharide and lignin composition analysis

*Asparagus* spear sections were separately ground with a mortar and pestle in liquid nitrogen and washed successively with 80% ethanol, acetone and methanol to generate alcohol insoluble residues (AIR) as described by Pettolino et al. [38]. The neutral sugar (alditol acetates) and polysaccharide (linkage analysis) compositions of the AIR were determined by GC-MS (Hewlett-Packard HP 6890 GC with a Hewlett Packard 5973 MS, Agilent) following the procedure described by Pettolino et al. [38].

Lignin content was determined gravimetrically as described by Luo et al. [39]. The final lignin-containing residue was weighed and the Klason-lignin content recorded as grams per 100 g FW.

### Preparation of microsomal membranes

Microsomes were isolated from *Asparagus* spears following the procedure of Zeng et al. [40] with minor modifications. Briefly, plant tissues were homogenized with extraction buffer (1 mL/g tissue) containing 50 mM HEPES-KOH (pH 6.8), 0.4 M sucrose, 1 mM dithiothreitol (DTT), 5 mM MnCl_2_, 5mM MgCl_2_ and complete EDTA-free proteinase inhibitor cocktail tablet (Roche, Basel, Switzerland) in a kitchen magic bullet blender. The homogenate was filtered through two layers of miracloth (Merck Millipore, Billerica, MA, US) and the filtrate was centrifuged at 3000g for 20 min at 4°C. The supernatant was centrifuged at 100,000g for 30 min at 4°C. The 100,000g pellets were resuspended in homogenization buffer and stored at -80°C. The protein concentration was measured using the BCA protein assay kit (Thermo Scientific, Rockford, IL, US). The bovine serum albumin (BSA) provided in the kit was used as the standard.

### Assay of xylan XylT activity by radiolabeling

Xylan XylT activity was measured according to Lee et al. [28]. Microsomes (300 μg of protein) were incubated in a 50 μL reaction mixture containing 50 mM HEPES-KOH (pH 6.8), 0.4 M sucrose, 1 mM DTT, 5 mM MnCl_2_, 5mM MgCl_2_ and 1% Triton-100, 0.5 mM Xyl_6_ and 1.4 μM UDP-[^14^C]-Xyl (total 15,000cpm) (PerkinElmer, Waltham, CA, US) and 0.25 mM unlabeled UDP-Xyl (CCRC, Athens, Georgia, US). After incubation at RT for 1 hr, the reaction was stopped by adding acetic acid and EGTA (final concentration 0.3 M and 20 mM, respectively). The radiolabeled xylo-oligosaccharides were separated from UDP-[^14^C]-Xyl by paper chromatography as described by Ishikawa et al. [41]. The terminated reaction mixture was spotted onto a strip of Whatman 3MM paper and developed overnight with the solvent of 95% ethanol: 1 M ammonium acetate (2: 1, v/v) as mobile phase. The radiolabeled Xyl-oligosaccharide products were immobilized on the baseline of the chromatogram and the radioactivity was counted with a Tri-Carb 2100TR liquid scintillation counter (PerkinElmer, Waltham, CA, US) using 4 mL of non-aqueous biodegradable counting scintillant (Amersham Biosciences, Pittsburgh, PA, US). Enzyme assays were performed at least in duplicate.

### Anthranilic acid labeling of xylo-oligosaccharides

The assay Xyl and xylo-oligosaccharides (Xyl_2_-Xyl_6_, Megazyme, Wicklow, Ireland) were labeled at their reducing termini with anthranilic acid (AA) according to the method described by Alwael et al [42]. Excess derivatisation reagent was removed by mixing with diethyl ether (x3) and the AA-labeled oligosaccharides (Xyl_1-6-_AA) in the bottom layer were purified using a Sep-Pak C18 cartridge (Waters, Milford, MA, US).

### Assay of xylan XylT activity using Xyl_n_-AA as acceptor and RP-HPLC analysis of Xyl_n_-AA products

The assay of XylT activity with Xyl_n_-AA acceptors was performed in a reaction mixture containing 50 mM HEPES-KOH (pH 6.8), 1 mM DTT, 5 mM MnCl_2_ and 1% Triton-100, 0.25 mM cold UDP-Xyl, 0.1 mM Xyl_n_-AA and 600 μg of microsomal membrane protein in a total volume of 100 μL. After incubation at RT, the reaction was terminated with acetic acid and EGTA (final concentration 0.3 M and 20 mM, respectively) and filtered through a 0.22 μm Ultrafree-MC filter (Merck Millipore, Billerica, MA, US). The products were analyzed by reversed-phase (RP) chromatography on an Agilent 1200 Series HPLC systems and JASCO FP-920 Intelligent Fluorescence Detector (Ex_320 nm_, Em_420 nm_). The Xyl_n_-AA products were separated on 2.1 mm × 100 mm, 1.8 μm ZORBAX Eclipse XDB-C_18_ RP column at a flow rate of 0.5 mL min^-1^ and a column temperature of 22°C using a gradient program set to: 0 to 2 min (8% B), 2.1 to 20 min (8% to 20% gradient B). Mobile phase A was 50 mM sodium acetate buffer (pH 4.3) and mobile phase B was acetonitrile.

### Gel filtration of xylan XylT assay products

The products of the radiolabelling XylT activity (as described above) were mixed with 0.5 ml 50% (v/v) Dowex 1x8 chloride form in water (Sigma-Aldrich, St. Louis, MO, US) and filtered through a 0.8 mL Pierce Centrifuge Tube (Thermo Scientific, Rockford, IL, US). The eluent was fractionated on a Bio-Gel P2 column (Bio-Rad, Hercules, CA, US) and fractions (1.8 ml) were collected. The radioactivity of each fraction (600 μl) was counted with a Tri-Carb 2100TR liquid scintillation counter (PerkinElmer, Waltham, CA, US) 4 mL of biodegradable aqueous scintillant (Amersham Biosciences, Pittsburgh, PA, US).

### Treatment of xylo-oligosaccharides with endo-xylanase and β-xylosidase

The XylT assay using the Xyl_n_-AA acceptors was terminated by heating at 100°C for 3 min. The products were then centrifuged at maximum speed in a microfuge for 5 min. Aliquots of the supernatant (10 μL) were saved for treatment with 10units of end-β-(1,4)-xylanase and 10 units of β-xylosidase by mixing with 30 μL 50 mM sodium acetate (pH 5.5,) containing either endo-β-(1,4)-xylanase (*Trichoderma viride*, GH11(M1), Megazyme, Wicklow, Ireland) or β-xylosidase (Sigma-Aldrich, St. Louis, MO, US), and incubated for 6 hr at 37°C. Digestions were terminated with 0.3 M acetic acid and subjected to RP-HPLC analysis as outlined above.

### MALDI-TOF-MS

The AA-labeled xylo-oligosaccharides collected from RP-HPLC were concentrated by SpeedVac and ZipTip (Millipore, Bedford, MA, US). The samples were analyzed on a 7T SolariX hybrid ESI/MALDI-FT-ICR-MS (Bruker, Billerica, Massachusetts, US). Aqueous sample (0.5 μl) was mixed (1: 1, v/v) with the MALDI matrix (saturated 2, 5-dihydroxbenzoic acid (DHB) in 50% acetonitrile) and dried on the stainless steel target plate. The MS was operated in the positive mode using the Broadband acquisition mode, averaging 8 spectra across the mass range = 247–1700 m/z, TD for acquisition was set to 1 M, source accumulation set to 0.001 s, ion accumulation set to 0.4 s and ion cooling set to 0.05 s. The laser spot size was set to large and laser power set at 30%, a total of 1000 laser shots were fired at a rate of 2 kHz per spectrum.

### RNA-Seq analysis of Asparagus spears and identification of candidate xylan synthesis genes

Total RNA from fresh and 4 day-old cold-stored *Asparagus* spears was extracted using the Isolate Plant RNA Mini Kit (Bioline, London, UK) and treated with DNase I (Invitrogen, Carlsbad, California, US) according to the manufacturer’s instructions to remove genomic DNA contamination. Total RNA samples were then quantified using a Nano-Drop (Thermo Scientific, Rockford, IL, US) and 10 μg total RNA submitted to AGRF (Melbourne, Victoria, Australia) for library construction (without size selection) and sequencing on a HiSeq 2000 (Illumina). Raw RNA-seq data from a single lane (191,179,245 read paired-end reads) was subjected to a quality control step involving adaptor removal and sequence trimming using Nesoni (http://www.vicbioinformatics.com/software.nesoni.shtml, min. length 30, quality 24, first 10 bp trimmed) and then *de novo*-assembled using Trinity [43]. The assembly was then scaffolded using CAP3 [44] (default settings) for each Trinity component.

A quantitative RNA-Seq experiment was also conducted using four biological replicates of apical, middle and basal section from fresh *Asparagus* spears, each replicate comprising tissue sections from five separate spears. Samples were run on an Illumina HiSeq 2000 in two lanes in a single flow-cell. All samples were sequenced in both lanes. Reads from each sample from each lane were merged and then filtered for quality and adaptor removal as per the reference sample. Reads for each sample were then quantitated independently against the reference transcriptome using RSEM v1.2.8 [45]. Orphaned reads were not used for quantitation. EBSeq v1.1 [46] was used to identify differentially expressed genes.

Candidate xylan biosynthetic genes and several known secondary cell wall regulation genes were identified in the *Asparagus* reference transcriptome by BLASTX searches (NCBI BLAST+ v2.2.25) using *Arabidopsis* sequences as bait.

### Sequence alignment and phylogenetic analysis

Sequence alignments and phylogenetic analysis was performed using Clustal X 2.1 [47]. IRX proteins from *Arabidopsis thaliana* (At), *Asparagus officinalis* (Ao), rice (*Oryza sativa*, Os) and moss (*Physcomitrella patens*, Pp) were aligned using Clustal X 2.1 and used as input to produce a neighbor-joining tree with 1000 bootstrap replicates using default settings.

### Semi-quantitative RT-PCR

One microgram of total RNA from each section was reverse-transcribed to cDNA using Tetro cDNA synthase kit (Bioline, London, UK) as per the manufacturer’s instructions. Semi-quantitative PCR was performed using My-Taq red DNA polymerase (Bioline, London, UK) with 1 μl cDNA as template. Thirty cycles of the following amplification program was used: 95°C 15 sec, 55°C 15 sec, 72°C 10 sec. PCR primer sequences are listed in S2 Table.

## Results

### Changes in fresh weight (FW), visual quality, texture and lignin content of garden asparagus spears after harvest

**Fig 1:**
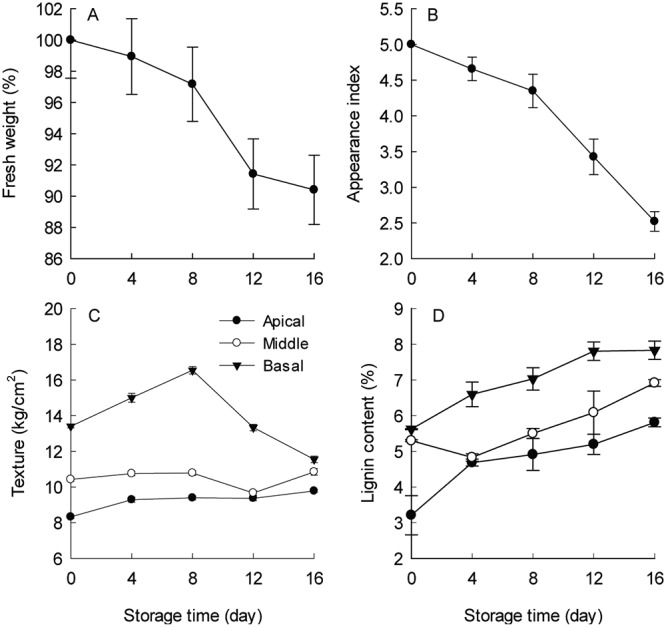
Physiological characteristics of fresh and stored *Asparagus* spears during low temperature (4°C) storage. (A) Changes in fresh weight, (B) visual appearance index, (C) texture (firmness) and (D) lignin content. The data are the average of fifteen *Asparagus* spears.

Green *Asparagus* spears stored at 4°C post-harvest had a reduction in FW of ~10% after 16 days of storage time (Fig 1A). The visual appearance index also dropped significantly (~50%; Fig 1B), correlating with the loss of FW. No statistically significant difference in wet weight was observed between 12–16 days of storage. However, during this time the appearance index dropped from ~3.5 (commercially acceptable) to ~2.5 (not commercially acceptable). Spear texture remained largely unchanged in the apical and middle sections (Fig 1C). Interestingly, the basal sections of the *Asparagus* spears had increased texture within the first 8 days of storage which then dropped between 8–16 days of storage. Lignin content of *Asparagus* spears steadily increased over storage time (Fig 1D) with the highest content in the basal section where the spear diameter is the largest, while the apical segment of the spear had the least lignin. These observations are consistent with previous reports [48].

### Changes in cell wall components in garden asparagus spears after harvest

The polysaccharide composition of different spear regions (apical, middle and basal) was determined by linkage analysis (summarized in Table 1, see also S1 Table). The major cell wall components of *Asparagus* spears include pectic arabinan, heteroxylan, xyloglucan (XG), heteromannan, cellulose and arabinogalactan (AG) (Type I and Type II) (Table 1). Strikingly, the different sections of the spear had different wall polysaccharide compositions. In fresh spears the apical section was composed mainly of arabinan (23%) and only modest amounts of heteroxylan (17%). In contrast, the basal section of the spear contained 55% heteroxylan, coincident with a harder texture and higher lignin content. Furthermore, the substitution levels of xylan, an indicator of solubility, were substantially lower in the basal section and increased from base to apex in both fresh and stored spears (S1 Table). The storage of *Asparagus* spears at 4°C for 16 days led to a significant decrease in the amount of arabinan, especially in the apical section of the spear where levels fell from 23% in fresh tissue to 18% in the stored tissue (Table 1). In contrast with arabinan, heteroxylan content increased in the apical and middle sections upon storage at 4°C, whereas there was no obvious change in the basal section. Heteromannan levels were similar and highest in the apical/middle sections for both fresh and stored spears. Interestingly, cellulose content in fresh apical and basal sections are lower to that of the middle section but upon storage these levels become similar over all sections.

**Table 1:** Cell wall neutral polysaccharide components (mol percent) in fresh and stored asparagus spears.

	Fresh			Stored		
	Bottom	Middle	Top	Bottom	Middle	Top
Arabinan	4	13	23	4	11	18
Type I AG	5	10	10	2	3	4
Type II AG	2	3	3	2	4	5
Glucuronoarabinoxylan	55	23	17	54	32	19
Heteromannan	6	13	11	8	15	15
Xyloglucan	5	7	6	3	5	8
Cellulose	12	20	14	19	17	18
Unassigned	12	11	16	8	13	13

### A comparison of xylan XylT activity among different plant species

Arabidopsis is an excellent system to study xylan biosynthesis via mutant analysis, however, biochemical investigation is challenging due to the limited availability of tissues and the inherently low activity of the xylan XylT in *in vitro* microsomal preparations. The *Asparagus* spear system was therefore examined for xylan XylT activity by comparison with existing models in dicots (*Arabidopsis*) and grasses (barley). Thus, a biochemical assay was optimized using microsomal membranes with either radiolabeled (UDP-[^14^C]-Xyl) or unlabeled UDP-Xyl and xylo-oligosaccharides (either free or the reductively aminated anthranilic acid (AA) derivatives).

**Fig 2:**
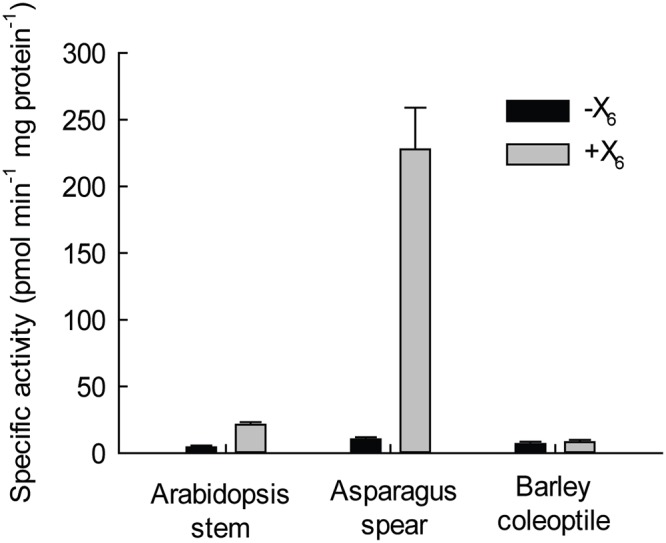
Comparison of xylan XylT activities in microsomes from *Arabidopsis*, *Asparagus* and barley (*Hordeum vulgare*). Microsomes from *Arabidopsis* stem, *Asparagus* spear and etiolated barley seedlings were isolated and the XylT activities were analyzed in the absence (-; black) and presence (+; grey) of the exogenous acceptor Xyl_6_ according to the Materials and Methods.

Using a paper chromatography assay measuring incorporation of [^14^C]-Xyl into a Xyl_6_ acceptor, *Arabidopsis* and particularly *Asparagus* microsomes showed XylT activity (Fig 2). In contrast, no activity could be detected in microsomes prepared from barley coleoptiles. *Arabidopsis* microsomes incorporated ~2% of total radiolabelled Xyl whereas *Asparagus* incorporated ~26%, i.e. a 13-fold higher amount than that of *Arabidopsis*.

### Biochemical properties of *Asparagus* xylan XylT activity

**Fig 3:**
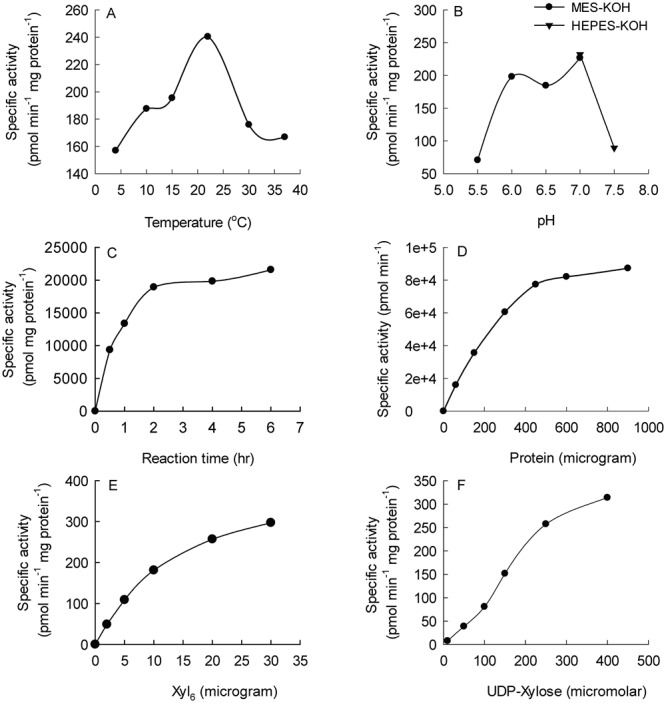
Biochemical properties of the xylan XylT activity in microsomes from garden asparagus spears. Microsomes extracted from the fresh basal *Asparagus* spears were incubated with UDP-[^14^C]-Xyl and Xyl_6_ for 1 hr unless otherwise indicated. The XylT activity was measured by counting the radioactivity at the origin of the paper chromatogram and expressed as specific activity. All assays were repeated twice and the values were averaged. Effect of temperature (A), pH (B), reaction time (C), microsomal protein amount (D), amount of Xyl_6_ (E) and UDP-Xyl concentration (F) on enzyme activity.

*Asparagus* xylan XylT activity was measured under standard radioactive assay conditions to be optimal at ~24°C and around pH 7 and (Fig 3A and 3B, respectively). The activity was time-, protein-, acceptor (Xyl_6_) and substrate (UDP-Xyl) concentration- (Fig 3C, 3D, 3E and 3F, respectively) dependent. The Michaelis-Menten constant (Km) values for Xyl_6_ and UDP-Xyl are 0.45 mM and 0.37 mM, respectively, as calculated from a Hanes-Woolf plot based on data presented in Fig 3E and 3F.

**Fig 4:**
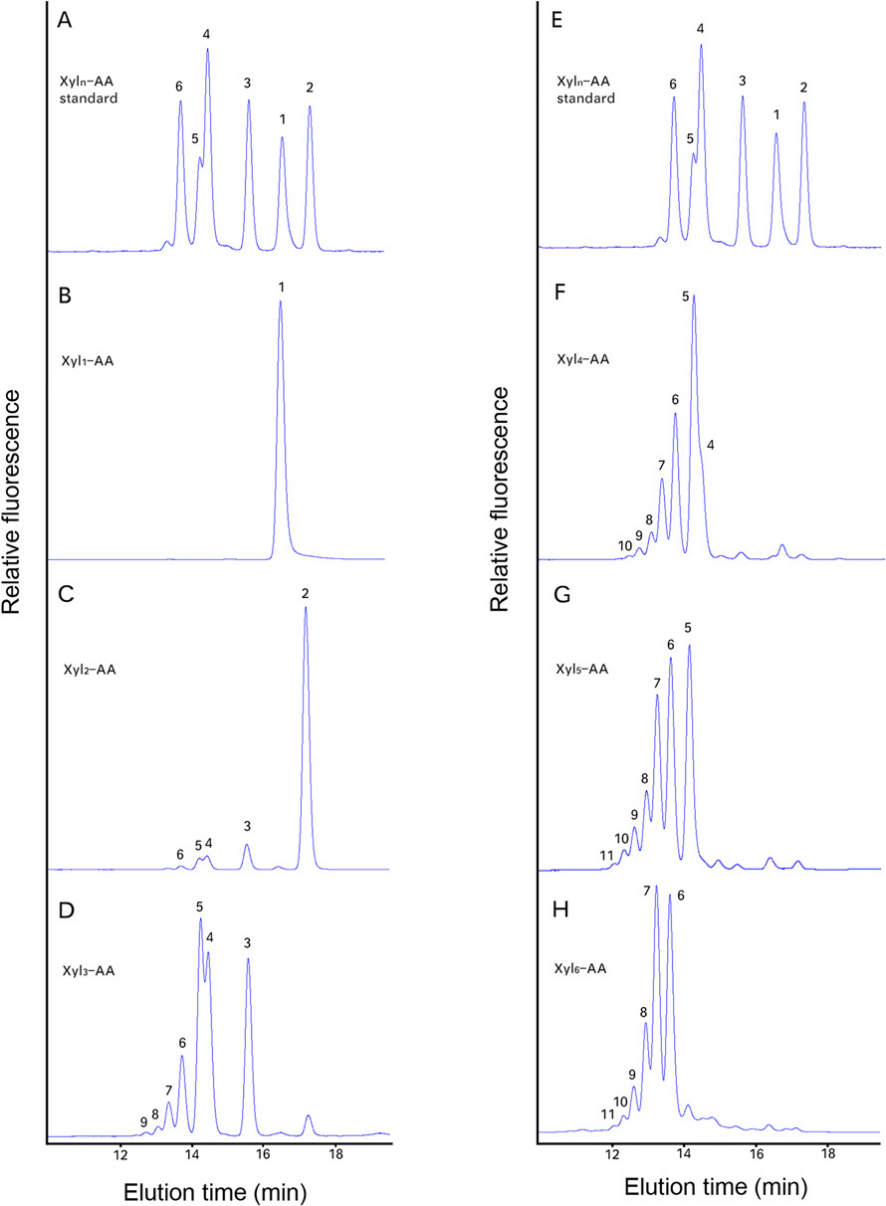
Xylan XylT activity measured using fluorescently tagged (AA) xylo-oligosaccharides of different lengths (Xyl_1_-Xyl_6_) as exogenous acceptors. The reaction was conducted by mixing *Asparagus* microsomes with cold UDP-Xyl and the fluorescent acceptors Xyl_1_-AA (B), Xyl_2_-AA (C), Xyl_3_-AA (D), Xyl_4_-AA (F), Xyl_5_-AA (G) or Xyl_6_-AA (H) and incubated at RT for 1 hr. The reaction products were separated by RP-HPLC and detected by a fluorescence detector. A chromatogram showing a separation of a Xyl_1_-AA to Xyl_6_-AA mixture of standards is shown in (A) and (E) for reference. The numbers on the plots indicates the DP of the xylo-AA oligosaccharides.

**Fig 5:**
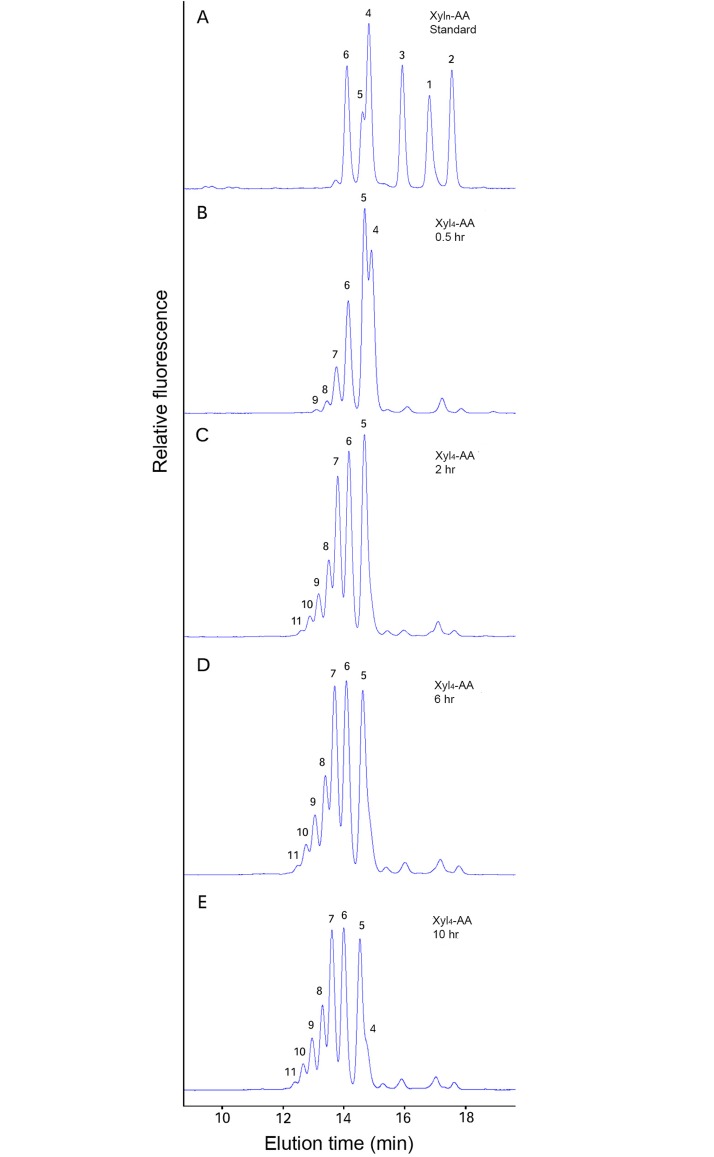
Time course of processive transfer of Xyl residues onto the Xyl_4_-AA acceptor. *Asparagus* microsomes were incubated with UDP-Xyl and the Xyl_4_-AA acceptor for 0.5 hr (B), 2 hr (C), 6 hr (D) and 10 hr (E). The reaction products were analyzed by RP-HPLC. (A) Standard chromatogram of Xyl_1_-AA to Xyl_6_-AA.

**Fig 6:**
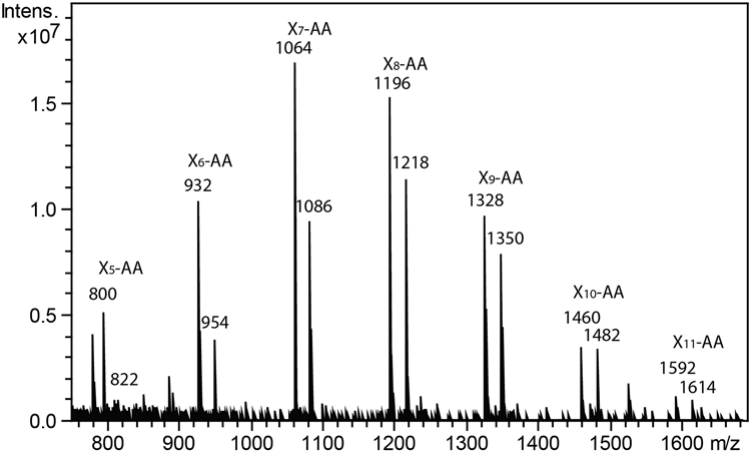
MALDI-TOF mass spectra of the xylan XylT reaction products catalyzed by *Asparagus* spear microsomes. Microsomes were incubated with UDP-Xyl and the Xyl_5_-AA acceptor for 1 hr. Reaction products were purified by RP-HPLC and the fraction between 12 and 15 min collected and analyzed by MALDI-TOF MS. The ions corresponding to AA-labeled xylo-oligosaccharides (both in H^+^ and Na^+^ form) are labelled.

**Fig 7:**
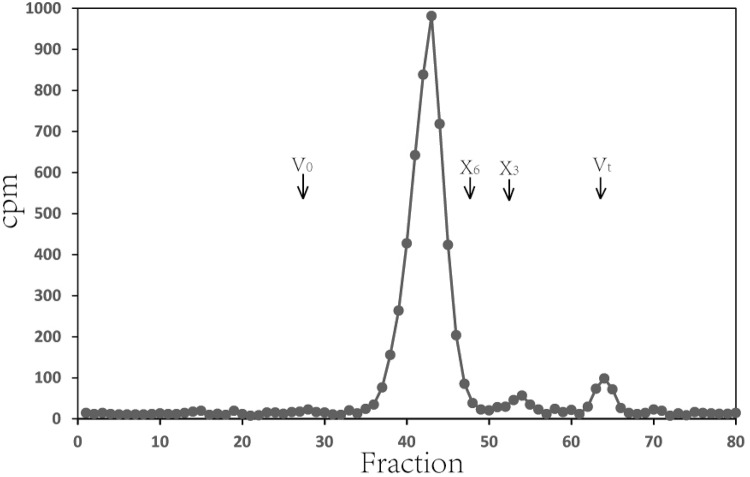
Enzymatic treatment of XylT reaction products generated using Xyl_4_-AA as acceptor. Microsomes were incubated with UDP-Xyl and the Xyl_4_-AA acceptor, and the reaction products were then digested with endo-β-(1,4)-xylanase (B) and β-xylosidase (C). The reaction products with (B, C) and without (A) enzymatic digestion were analyzed by RP-HPLC.

In order to more easily characterize the *Asparagus* products of the XylT activity assay, acceptor xylo-oligosaccharides were fluorescently tagged with anthranilic acid (AA) at their reducing end and used in a slightly modified XylT assay (see Materials and Methods). The assay products generated by *Asparagus* microsomes were then characterized by reversed-phase (RP)-HPLC (Figs 4 and 5), MALDI-TOF-MS (Fig 6) and enzyme digestion (Fig 7). Interestingly, the Xyl_1_-AA and Xyl_2_-AA were eluted in the “opposite” order to the rest of AA labeled xylo-oligosaccharide standards (DP3-6) (Fig 4A), which eluted faster with increasing DP. This order of the AA labeled xylo-oligosaccharide standard was confirmed by RP-HPLC-MS analysis (data not shown). No activity was detected with the monomer (Xyl_1_-AA) as the acceptor (Fig 4B). The *Asparagus* XylT activity increased with higher DP (2–6) of the acceptors (Fig 4C, 4D, 4F, 4G and 4H, respectively).

Lee et al. [10]) demonstrated that in *Arabidopsis* a longer reaction time could lead to the production of higher DP xylo-oligosaccharides by the XylT. We also performed a time course study using Xyl_4_-AA as the acceptor (Fig 5) and found up to seven Xyl residues were added within a 2 hr incubation period, resulting in xylo-oligosaccharide products ranging from DP5-11 (Fig 5C). Longer incubation times, up to 10 hr, did not result in the formation of longer xylo-oligosaccharides (Fig 5).

The products of the XylT activity using Xyl_5_-AA as the acceptor were characterized by MALDI-TOF-MS. The observed molecular ion series (protonated and sodiated adducts) corresponded to the expected molecular masses for a pentitol series (+132 a.m.u) of oligosaccharides of DP 5–11 (m/z 800, 932, 1064, 1196, 1328, 1460, 1592 and 822, 954, 1086, 1218, 1350, 1482, 1614, respectively) series, corresponding to the H^+^ and Na^+^ forms, respectively (Fig 6).

**Fig 8:**
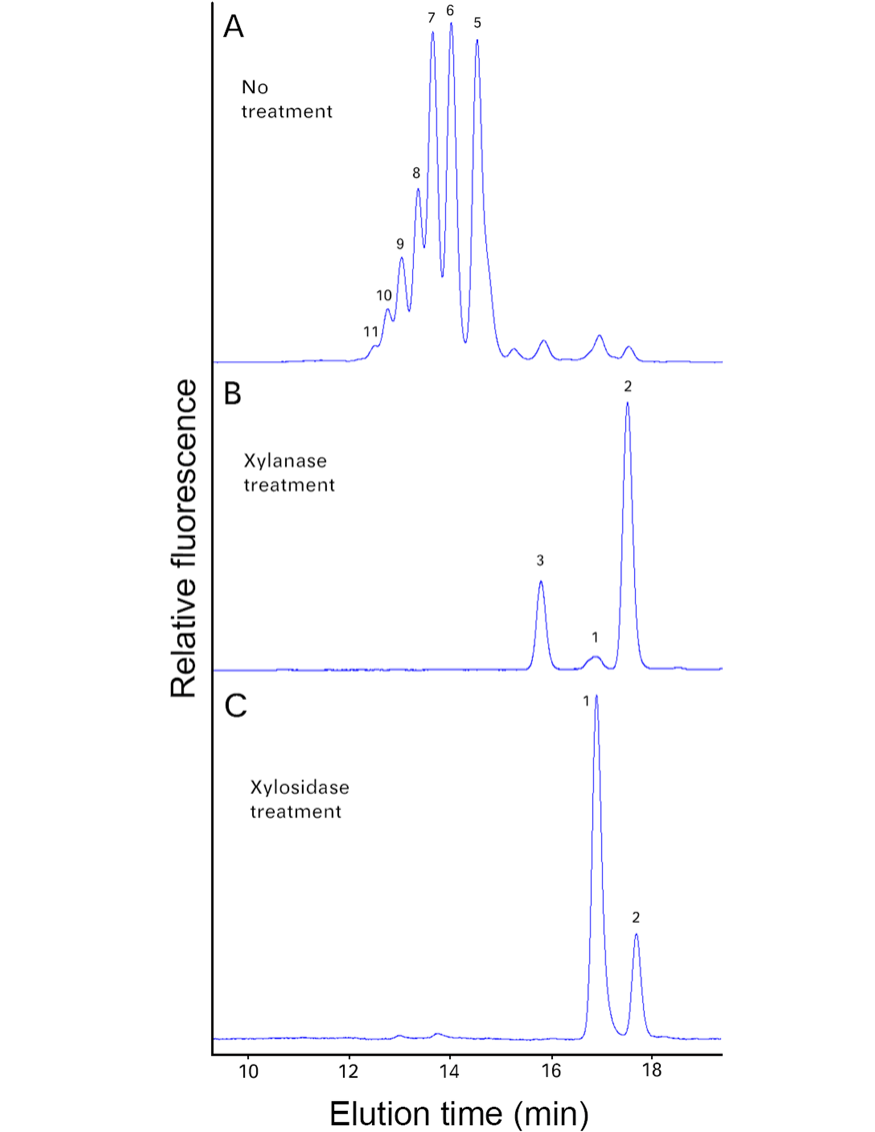
XylT reaction products generated using Xyl_6_ as the acceptor were fractionated on a Bio-Gel P2 column. *Asparagus* microsomes were incubated with UDP-[^14^C]-Xyl and Xyl_6_ acceptor. The reaction products were treated with Dowex resin to remove unreacted UDP-[^14^C]-Xyl and fractionated on Bio-Gel P2 column. V_0_ and Vt, void and total volume, respectively. X_6_ and X3, xylo-hexaoligosaccharide and xylo-trioligosaccharide standards.

The products of the XylT reaction generated using Xyl_6_ as the acceptor and UDP-[^14^C]-Xyl as the donor were fractionated on a Bio-Gel P2 column (Fig 8). Around 5000 cpm of [^14^C]-Xyl product was loaded onto the column and the majority (96%) of the products were eluted between fraction 37 and 47, slightly earlier than the Xyl_6_ standard. There were two minor peaks at fractions 54 and 64 (V_t_) corresponded to Xyl_3_ and Xyl_1_ respectively.

The linkage/anomeric configuration of the xylo-oligosaccharide products was then established by enzymatic hydrolysis using xylan-specific enzymes, namely an endo-β-(1,4)-xylanase and a β-xylosidase. The endo-xylanase degraded the xylo-oligosaccharides to DP2 and DP3 whereas the β-xylosidase degraded the xylo-oligosaccharides to DP1 and some DP2 (Fig 7), confirming that the Xyl is linked β–(1,4).

### A comparison of the xylan XylT activity in garden asparagus spears during post-harvest cold storage

**Fig 9:**
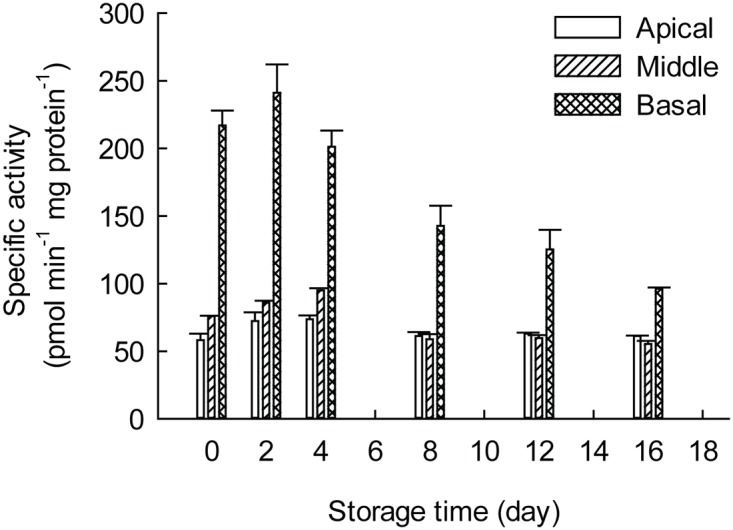
Xylan XylT activities of different *Asparagus* sections over the 4°C storage period. Microsomes from apical, middle and basal sections of *Asparagus* spears stored at 4°C for 0–16 days were isolated and the XylT activities were measured as described in the Materials and Methods. Data were average values±SE (n = 3).

To investigate changes in xylan XylT activity during the storage process, *Asparagus* spear tissues from six stages (0, 2, 4, 8, 12, 14 days post-harvest) were collected and the XylT activity of the apical, middle and basal sections was analyzed. The basal tissue was found to have four times higher XylT activity than the apical section in freshly harvested tissues, correlating with the increased heteroxylan content of this section compared to the apex (Fig 9). Notably, the XylT activity slightly increased during the initial stage of storage (0–2 day after harvest) and then declined in all three sections with additional storage time (Fig 9).

The *Asparagus* spear offers distinct advantages to study the biochemical/molecular mechanism(s) of xylan biosynthesis with its vastly superior xylan XylT activity compared to other plant models, yet *Asparagus officinalis* lacks a sequenced genome. We therefore decided to use an RNA-Seq approach to identify the genes involved in xylan biosynthesis in this system. In an initial experiment, a combined sample of total RNA extracted from fresh and 4 day-old cold-stored *Asparagus* spears was sequenced. The raw RNA-Seq dataset comprised 191,179,245 paired-end reads. After quality trimming and adapter removal, a total of 174,487,864 read pairs remained and were assembled into 403,090 contigs (N50 47503, L50 1824bp, 293.4Mb). The assembly contigs were then scaffolded [44] which resulted in a reference transcriptome consisting of 351,350 transcript isoforms.

BLASTX searches were used to identify the putative *Asparagus* orthologs of known *Arabidopsis* xylan synthesis genes including GTs (*IRX9*, *IRX10*, *IRX14*, *FRA8*, *F8H*, *IRX8*, *PARVUS*, *GUX1*) and non-GTs (*UXS*, *GXM*, *IRX15*, *TBL29*). Other secondary wall biosynthesis genes including phenylalanine ammonia-lyase *(PAL)* [49] and the NAC secondary wall thickening promoting factor-1 (*NST1*) [50] representing proteins involved in lignin biosynthesis and a secondary wall transcription factor, respectively, were also searched within the reference transcriptome. As the focus of this study was on xylan XylT activity, *IRX9*, *IRX10* and *IRX14* and their related homologs were chosen for further analysis. The full-length open reading frames of five XylT genes were identified and designated *AoIRX9*, *AoIRX9-L*, *AoIRX10*, *AoIRX14_A*, *AoIRX14_B* on the basis of their similarity to the *Arabidopsis* sequences. The corresponding contigs are listed in Table 2. *IRX9* and *IRX14* belong to CAZy family GT43 (http://www.cazy.org/) and a phylogenetic tree was created using *Arabidopsis* and *Asparagus* GT43 protein sequences (S1 Fig). Both *Arabidopsis IRX9* and *IRX-9L* have apparent orthologs in *Asparagus*, although it is uncertain which *Asparagus* sequence is the true ortholog in the case of *IRX-14_A/14_B*, hence their generic naming. Interestingly, only one *IRX10* gene was found in the *Asparagus* transcriptome which shares 86% and 89% identity with *AtIRX10* and *AtIRX10-L*, respectively (S2 Fig). The full-length sequences of these genes have been deposited in the Genbank database (http://www.ncbi.nlm.nih.gov/Genbank) with the following accession numbers: *AoIRX9* (KJ556998); *AoIRX9-L* (KJ556999); *AoIRX10* (KJ557000); *AoIRX14_A (*KJ557001); *AoIRX14_B* (KJ557002).

**Table 2:** *Asparagus* orthologs of *Arabidopsis* xylan biosynthetic genes as well as GT2 (cellulose synthase and cellulose synthase like genes) and representatives of known secondary wall associated genes.

*Asparagus* contig no.	AGI	Gene Name	Proposed Function	E-Value	Stem section		
					Apical	Middle	Basal
comp82040_c0	AT2G37090	*IRX9*	backbone synthesis	e-103	0.17±0.02	9.93±0.32	13.73±0.18
comp85515_c0	AT1G27600	*IRX9-L*	backbone synthesis	e-105	55.13±1.48	48.58±2.48	38.76±1.52
comp90084_c0	AT1G27440	*IRX10*	backbone synthesis	0	40.05±2.15	44.87±1.67	43.43±0.77
comp84164_c0	AT5G67230	*IRX14-L*	backbone synthesis	e-131	19.26±1.23	21.90±0.59	22.1±1.11
comp86147_c0	AT5G67230	*IRX14-L*	backbone synthesis	e-102	14.47±0.98	23.61±0.86	19.82±0.34
comp89578_c0	AT2G28110	*IRX7*	reducing end synthesis	e-145	28.67±1.02	35.87±0.89	52.76±2.76
comp84659_c0	AT5G22940	*IRX7-L*	reducing end synthesis	0	1.84±0.10	3.75±0.26	3.89±0.09
comp29843_c0	AT5G54690	*IRX8*	reducing end synthesis	0	5.64±0.40	6.67±0.34	7.86±0.54
comp74252_c0	AT5G54690	*IRX8*	reducing end synthesis	0	7.23±0.35	7.35±0.62	8.87±0.66
comp88939_c1	AT1G19300	*PARVUS*	reducing end synthesis	e-155	40.79±2.25	45.9±4.44	57.07±4.28
comp84705_c0	AT3G18660	*GUX1*	GlcA incorporation	0	18.46±0.70	14.39±0.82	15.87±0.36
comp109454_c0	AT3G18660	*GUX1*	GlcA incorporation	e-122	2.25±0.76	4.36±1.09	5.06±0.71
comp79489_c0	AT3G62830	*UXS2*	UDP-Xyl synthase	0	75.6±3.86	51.41±3.32	45.58±2.40
comp61026_c0	AT3G53520	*UXS1*	UDP-Xyl synthase	e-171	53.58±1.50	53.11±1.86	59.72±0.83
comp37556_c0	AT1G09610	*GXM1*	GlcA methyltransferase	3e-91	2.69±0.30	11.6±0.58	10.82±0.72
comp60971_c0	AT5G67210	*IRX15-L*	unknown	4e-67	10.15±1.77	4.1±0.41	4.26±1.08
comp80607_c1	AT3G55990	*TBL29*	acetyltransferase	0	0.18±0.05	1.5±0.18	1.07±0.23
comp67577_c0	AT3G55990	*TBL29*	acetyltransferase	e-158	0.52±0.12	2.13±0.46	1.66±0.21
comp91651_c0	AT4G32410	*CESA1*	cellulose synthase	0	13.31±0.76	15.80±1.42	13.35±1.26
comp82725_c0	AT5G05170	*CESA3*	cellulose synthase	0	4.16±0.41	4.31±0.29	4.24±0.33
comp88863_c0	AT5G44030	*CESA4*	cellulose synthase	0	27.06±1.32	43.67±0.96	39.05±1.97
comp86858_c0	AT5G09870	*CESA5*	cellulose synthase	0	0.82±0.18	1.70±0.22	1.84±0.25
comp90588_c1	AT5G64740	*CESA6*	cellulose synthase	0	230.2±6.44	291.1±35.4	247.9±36.6
comp43060_c0	AT5G17420	*CESA7*	cellulose synthase	0	0.39±0.11	11.76±0.51	13.91±0.69
comp76272_c0	AT5G03760	*CSLA9*	mannan synthase	0	11.77±0.26	13.84±1.25	16.24±0.90
comp78814_c0	AT5G03760	*CSLA9*	mannan synthase	0	10.03±0.54	10.33±0.47	14.93±0.81
comp78728_c1	AT5G03760	*CSLA9*	mannan synthase	e-175	13.59±0.15	8.01±0.38	14.39±0.81
comp187879_c0	AT5G03760	*CSLA9*	mannan synthase	0	0.28±0.10	1.14±0.36	0.95±0.21
comp80676_c0	AT4G07960	*CSLC12*	xyloglucan synthase	0	30.20±1.48	18.71±1.78	17.57±0.52
comp83916_c0	AT3G03050	*CSLD3*	cellulose synthase	0	17.64±1.00	16.55±2.02	18.69±1.66
comp87435_c0	AT1G55850	*CSLE1*	unknown	0	17.78±1.30	16.73±1.35	24.04±1.52
comp89194_c0	AT1G55850	*CSLE1*	unknown	e-179	34.80±0.84	32.19±1.61	29.54±2.44
comp81970_c0	AT4G23990	*CSLG3*	unknown	e-179	13.95±0.39	18.46±0.86	26.05±1.82
comp86723_c0	AT2G37040	*PAL*	lignin synthesis	0	28.09±1.01	41.67±2.48	127.73±2.78
comp74161_c0	AT2G46770	*NST1*	secondary wall TF	2e-84	0.33±0.12	2.53±0.38	3.54±0.43
comp59563_c0	AT5G09810	*ACTIN*	house keeping	0	936.3±15.3	943.1±25.7	844.1±31.6

Gene expression in the basal, middle and apical sections is shown as transcripts per million (TPM) ± SD (n = 4). The house keeping gene *ACTIN* is used a control.

A separate quantitative RNA-Seq experiment was also conducted using four biological replicates of apical, middle and basal section from fresh *Asparagus* spears. The relative expression values (transcripts per million reads, TPM) of the five *Asparagus* putative xylan backbone XylT genes in the basal, middle and apical stem sections are listed in Table 2. *AoIRX9-L*, *AoIRX10*, *AoIRX14_A* and *AoIRX14_B* do not display differential expression across the spear sections, in contrast to *AoIRX9* which shows substantially higher expression in the middle and basal stem sections compared to the apex. This expression pattern is similar to *AoPAL* and *AoNST1* whose expression is higher in the base of the stem where more secondary wall synthesis is occurring.

The expression of the five xylan backbone XylT genes was also analyzed by semi-quantitative RT-PCR to confirm the pattern and relative transcript abundance observed in RNA-Seq analysis. The expression of *AoIRX9* in the basal and middle sections was again observed to be higher than that of the apical section while other xylan backbone genes show no differential expression throughout all three sections of the spear (S3 Fig). As expected, *PAL* and *NST1* were more highly expressed in the basal compared to the apical section. In all cases, the pattern and intensity of the amplified PCR product correlated well with the RNA-Seq quantitation results, providing robust validation of these data (Table 2).

## Discussion

### *Asparagus* spears undergo significant physiological and biochemical changes after harvest, including increased lignification

As an immature and fast growing stem tissue, *Asparagus* spears retain high physiological activities including high respiration rates, the depletion of soluble sugars, proteins and ascorbic acid, and pronounced water losses after harvest and cold storage in the dark [36, 51]. In the present study, the spears rapidly lost water content and their visual appearance was reduced to the point of being unattractive to consumers after 16 days of storage at 4°C (Fig 1A and 1B). The more mature basal part of the fresh spear had a harder texture and higher content of lignin than the immature apical part (Fig 1C and 1D). The continuation of shoot differentiation after harvest results in secondary wall thickening and increased lignification. However, the process of hardening does not take place uniformly along the length of the spear due to differing respiration rates [36]. Significant increases in lignin levels were found in all three sections upon cold storage, with the greatest increase observed in the basal section (Fig 1D), indicating cold treatment-induced lignification of garden asparagus spears. However, there were no significant changes in texture in either the middle or apical sections during cold storage, whereas the texture of the basal section was the greatest in the sections at day 0, and increased until day 8 of storage and then decreased to the day 0 level by day 16 (Fig 1C). These results are consistent with those described by Villanueva-Suarez et al. [52] who suggested the hardening process was related to wall polysaccharide changes and water loss as well as the amounts of lignin. Further, our data (see Discussion below) would suggest that the process likely also involves the relative lowly substituted heteroxylans that increase significantly during this period of storage (Table 1 and S1 Table) and which would be expected to self-associate to form “hydrophobic bundles” of insoluble polysaccharides that exclude water, much like cellulose microfibrils adding further rigidity to the wall[53,54].

### An *in-vitro* xylan XylT assay indicates *Asparagus* spears actively synthesize heteroxylan during storage at 4°C

Previous reports of xylan XylT activity have mostly focused on tissues of dicot and commelinoid monocot species undergoing normal growth and development but there is scant information about changes in XylT activity for non-commelinoid monocot species such as *Asparagus*. We detected a xylan XylT activity in the apical, middle and basal sections of *Asparagus* spears that increases during the early phase of cold storage after harvest and then decreases (Fig 9). The increased XylT activity was coincident with the lignification of *Asparagus* spears, suggesting a coordinated deposition of secondary wall polymers resulting in a rigidification of the spears. Using a biochemical assay based upon the incorporation of Xyl from UDP-Xyl into a xylo-oligosaccharide acceptor, we showed that *Asparagus* spear xylan XylT activity is significantly higher (thirteen fold) than in *Arabidopsis* stems (Fig 3). As a non-commelinoid monocot and different from grasses, *Asparagus* has a type-I cell wall composition similar to dicots [55,56].

We have demonstrated that the *Asparagus* XylT has processive activity capable of adding up to seven Xyl residues to a xylo-oligosaccharide acceptor of DP4 (see Fig 5) with kinetic characteristics (optimal pH, temperature, etc.) similar to those previous published in Arabidopsis [10] and wheat [27]. The K_m_ values of UDP-Xyl and pyridylaminated (PA) Xyl_5_ (Xyl_6_-PA was not analyzed) of etiolated wheat seedlings were 7.9mM and 2.9mM [27], respectively, higher than what was observed for the *Asparagus* microsomes. This correlates with the higher XylT activity of *Asparagus*. The *Km* value of Xyl_6_ (0.45 mM) is lower than previous published for the purified AtIRX10-L protein (1.17 mM) [21] but this could be due to a different combination of proteins in the xylan XylS in the biosynthetic protein complex of *Asparagus* compared to *Arabidopsis*. However, the *Asparagus* XylS is more amenable for purification due to its considerably higher activity thus assisting in studying its molecular mechanism of action.

The degree of polymerization (DP) of xylo-oligosaccharides synthesized by *Asparagus* microsomes *in vitro* (DP 7–11) is still far smaller than the native heteroxylan, which is between 150–200 residues [36]. It is generally assumed that the “xylan synthase (XylS)” *in vivo* is a protein complex comprising multiple GTs [5], although the mechanism that controls the length of the xylan chain and the termination of xylan biosynthesis is not well understood [5]. Based upon assays of heterologously expressed IRX proteins and *in vitro* biochemical assays, Urbanowicz et al [21] have proposed that the *Arabidopsis* IRX10-L is the catalytic xylan synthase (XYLAN SYNTHASE-1, XYL1) and whilst not ruling out a catalytic function for IRX 9/14 they propose that although these two proteins are a part of the xylan synthase complex their role is more likely in either chain initiation and/or a structural role in stabilizing the synthase complex.

### Next-generation sequencing provides a powerful tool to use *Asparagus* as a model to study heteroxylan biosynthesis

The recently developed RNA-seq technology is a powerful tool to study gene expression, especially for non-model species [57]. The *Asparagus* RNA-seq dataset makes a genome wide comparison between *Asparagus* and the model dicot *Arabidopsis* possible. It is not surprising that all the *Arabidopsis* xylan biosynthesis related genes have orthologs in *Asparagus* (Table 2). *Arabidopsis IRX9(-L)* and *IRX14(-L)* belong to the CAZy GT43 family. *Arabidopsis* mutant studies show that they are separate classes of two non-redundant genes both involved in xylan biosynthesis and that IRX9 and IRX14 play the major role while the redundant IRX9-L and IRX14-L function in a minor role [11]. Tobacco suspension cells co-expressing the *Arabidopsis IRX9* and *IRX14* have been shown to possess xylan backbone elongation activity but the activity is absent when either gene is expressed alone [28]. This suggests that IRX9 and IRX14 operate in a xylan biosynthesis protein complex. In grasses, rice IRX9 and IRX14 have been demonstrated to be involved in xylan biosynthesis via *Arabidopsis* mutant complementation analyses [58]. In this study, *AoIRX9* shows a distinct expression pattern compared with the other *IRX* genes. Its expression is significantly higher in the basal section than in the apical section in fresh *Asparagus*, and this correlates with the higher xylan XylT catalytic activity in the basal tissue. The semi-quantitative RT-PCR data is consistent with RNA-seq analysis of the gene expression in the basal, middle and apical sections of fresh *Asparagus* spears (Table 2 and S3 Fig). A site-directed mutagenesis study on *AtIRX9* has shown that IRX9 appears not to be directly involved in catalytic activity [59]. Combined with the quantitative RNA-seq analysis, this indicates that AoIRX9 is playing a key role in regulating xylan biosynthesis during secondary wall biosynthesis in *Asparagus*. For example, AoIRX9 may function by recruiting the other XylTs to form an active xylan synthase complex in the Golgi apparatus, consistent with the conclusions drawn by Urbanowicz et al [21].

Mutant analysis has revealed *Arabidopsis IRX10* and *IRX10-L* play an essential role in xylan biosynthesis. The two homologs are functionally redundant and double mutants show a severe growth phenotype and are also infertile [8,9]. Down-regulation of rice Os*IRX10* leads to decreased xylan and a moss *IRX10* could only partially complement the *Arabidopsis irx10 irx10-L double* mutant [60,61]. Urbanowicz et al [21] have provided biochemical evidence that AtIRX10-L (XYL1) is a xylan synthase while no XylT was demonstrated for AtIRX9 and AtIRX14. Although IRX10 protein sequences are conserved from mosses to vascular plants (S2 Fig) and it remains unknown what role IRX10 plays in the non-vascular plant. Interestingly, only one *IRX10* gene was identified in the *Asparagus* spear transcriptome. A blast analysis of the *Asparagus densiflorus* transcriptome (1000 plants initiative, https://sites.google.com/a/ualberta.ca/onekp/) with *Arabidopsis* IRX10 also identified only one *IRX10* (data not shown), providing further evidence that there may only be one IRX10 gene in *Asparagus* species although sequencing of the *Asparagus* genome will be required to provide a definitive answer. It still remains a mystery as to how IRX9, IRX10 and IRX14 interact with each other (and other GTs and the *O*-acetyl transferase (XOAT1)) to coordinate heteroxylan backbone synthesis.

Genomic analysis suggests that the Asparagales are more similar to eudicots than to Poales [62]. Given most species in this order have large genomes, the genetic information currently available for these non-commelinoid monocots is limited. The family Asparagaceae has the smallest genome among the Asparagales and it has been proposed that *Asparagus* be used as a genomic model [63]. In this study, the transcriptome database provides an essential tool to study the gene expression and characterization of candidate genes/proteins associated with xylan synthesis in *Asparagus*.

## Conclusion

We have identified and characterized a highly active xylan XylT in *Asparagus officinalis*, a non-commelinoid monocot. This XylT activity correlates with increased xylan content and is the most promising plant model in which it is sufficiently active in *in vitro* microsomal preparations to enable purification of the native xylan synthase protein complex (XylS). Transcriptome analysis revealed five candidate XylT synthase genes which together with a functional XylT assay will allow us to establish their precise roles in xylan biosynthesis and shed new light on the mechanism of assembly.

## Supporting Information

S1 Fig Phylogenetic tree for the GT43 proteins in *Arabidopsis* and *Asparagus*. Four predicted *Asparagus* sequences (AoIRX9, AoIRX9-L, AoIRX14_A and AoIRX14_B) were aligned with *Arabidopsis* GT43 proteins using Clustal X 2.1 and a neighbor-joining tree was produced. The bar represents a percent accepted mutations value of 10%. The numbers shown at branching points are bootstrap values derived from 1000 randomized samples.
(TIF)

S2 Fig Sequence alignment of the *Arabidopsis*, *Asparagus*, rice and *Physcomitrella* IRX10 proteins. IRX10 sequences were aligned using Clustal X 2.1. Alignment shown in Jalview 2.8 where blue shading indicates sequence identity across the five sequences. Greater than 80% identity is dark blue, greater than 60% is medium blue, greater than 40% is light blue and 40% or less has no shading.
(TIF)

S3 Fig Semi-quantitative RT-PCR analysis of *Asparagus* XylT genes as well as two known secondary wall biosynthetic genes. Total RNA was extracted from apical, middle and basal sections of fresh *Asparagus* spears and the expression patterns of *AoIRX9*, *AoIRX9-L*, *A*oIR*X10*, *AoIRX14_A*, *AoIRX14_B*, *PAL* and *NST1*, were analyzed by semi-quantitative RT-PCR using actin as a control.
(TIF)

S1 Table Monosaccharide linkage analysis of AIR from *Asparagus* basal (B), middle (M) and apical (A) spears. (DOC)

S2 Table Real-time PCR primers of *Asparagus* xylan synthase and actin genes. (DOC)
