# Supplementary figures and images for: Asparagus Spears as a Model to Study Heteroxylan Biosynthesis during Secondary Wall Development
Source: PLoS One. 2015 Apr 20;10(4):e0123878. doi: 10.1371/journal.pone.0123878 (PMC4404143; doi:10.1371/journal.pone.0123878)

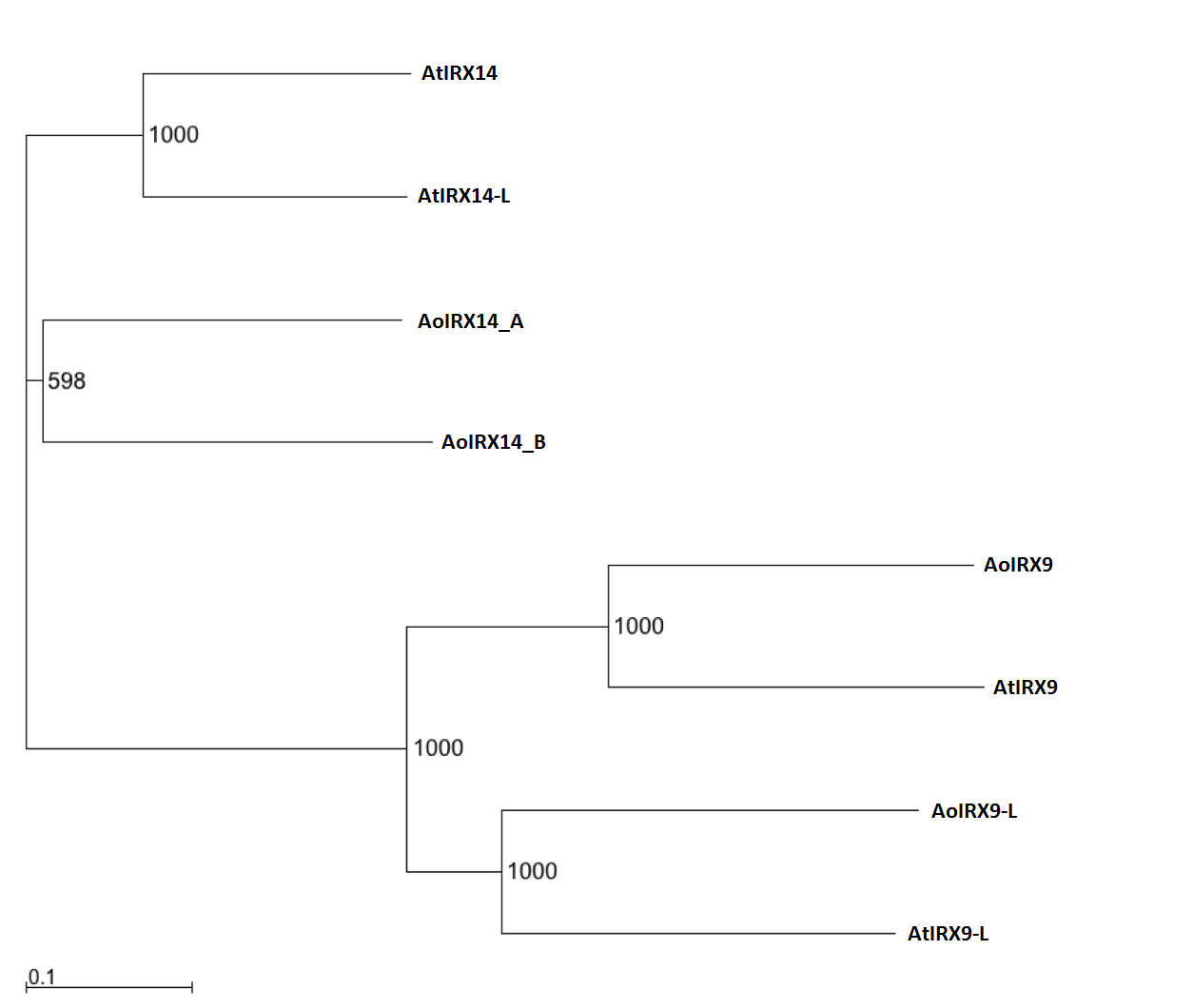

Supplement: S1 Fig — Four predicted Asparagus sequences (AoIRX9, AoIRX9-L, AoIRX14_A and AoIRX14_B) were aligned with Arabidopsis GT43 proteins using Clustal X 2.1 and a neighbor-joining tree was produced. The bar represents a percent accepted mutations value of 10%. The numbers shown at branching points are bootstrap values derived from 1000 randomized samples. (TIF) [file pone.0123878.s001.tif]

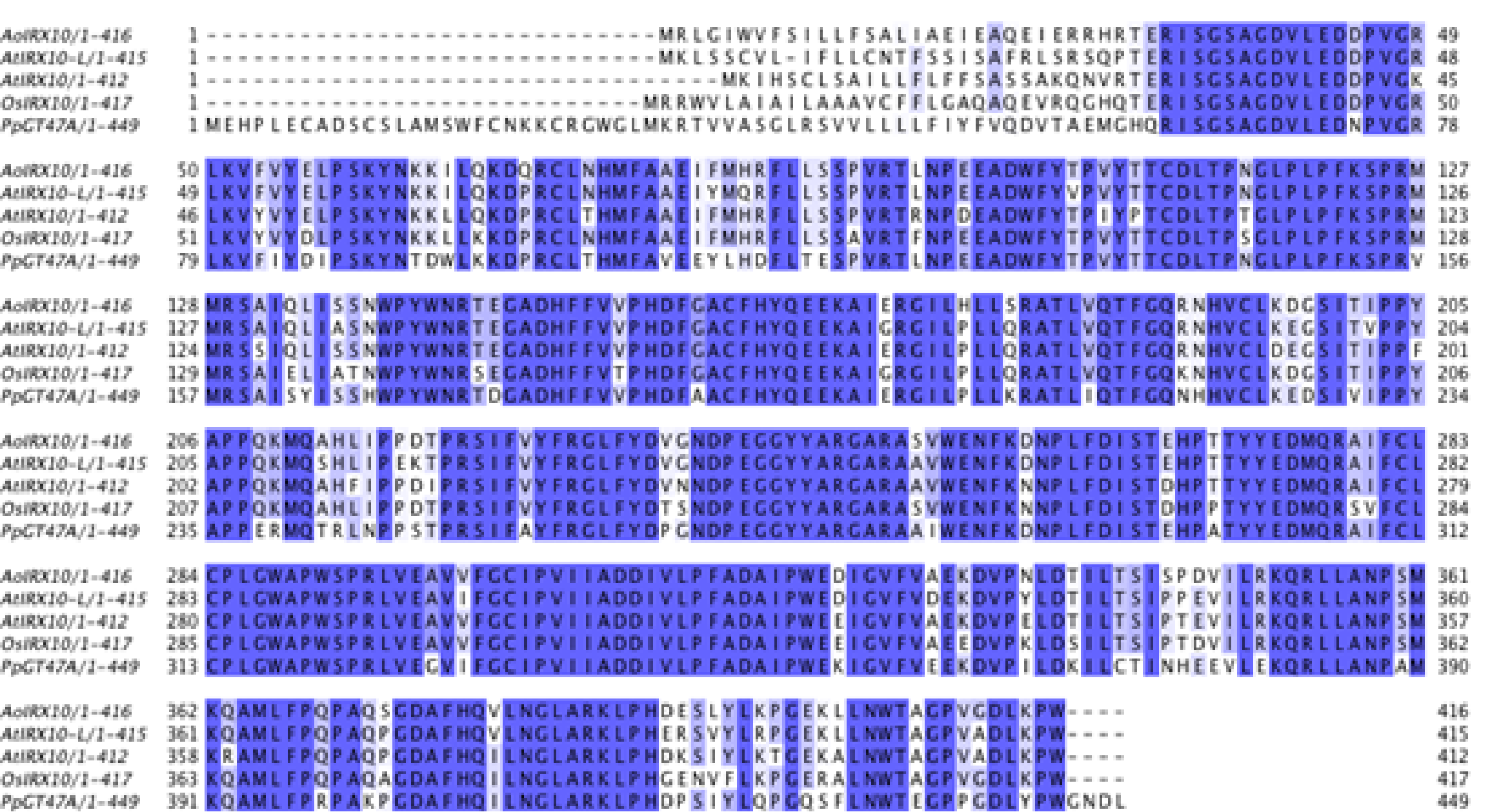

Supplement: S2 Fig — IRX10 sequences were aligned using Clustal X 2.1. Alignment shown in Jalview 2.8 where blue shading indicates sequence identity across the five sequences. Greater than 80% identity is dark blue, greater than 60% is medium blue, greater than 40% is light blue and 40% or less has no shading. (TIF) [file pone.0123878.s002.tif]

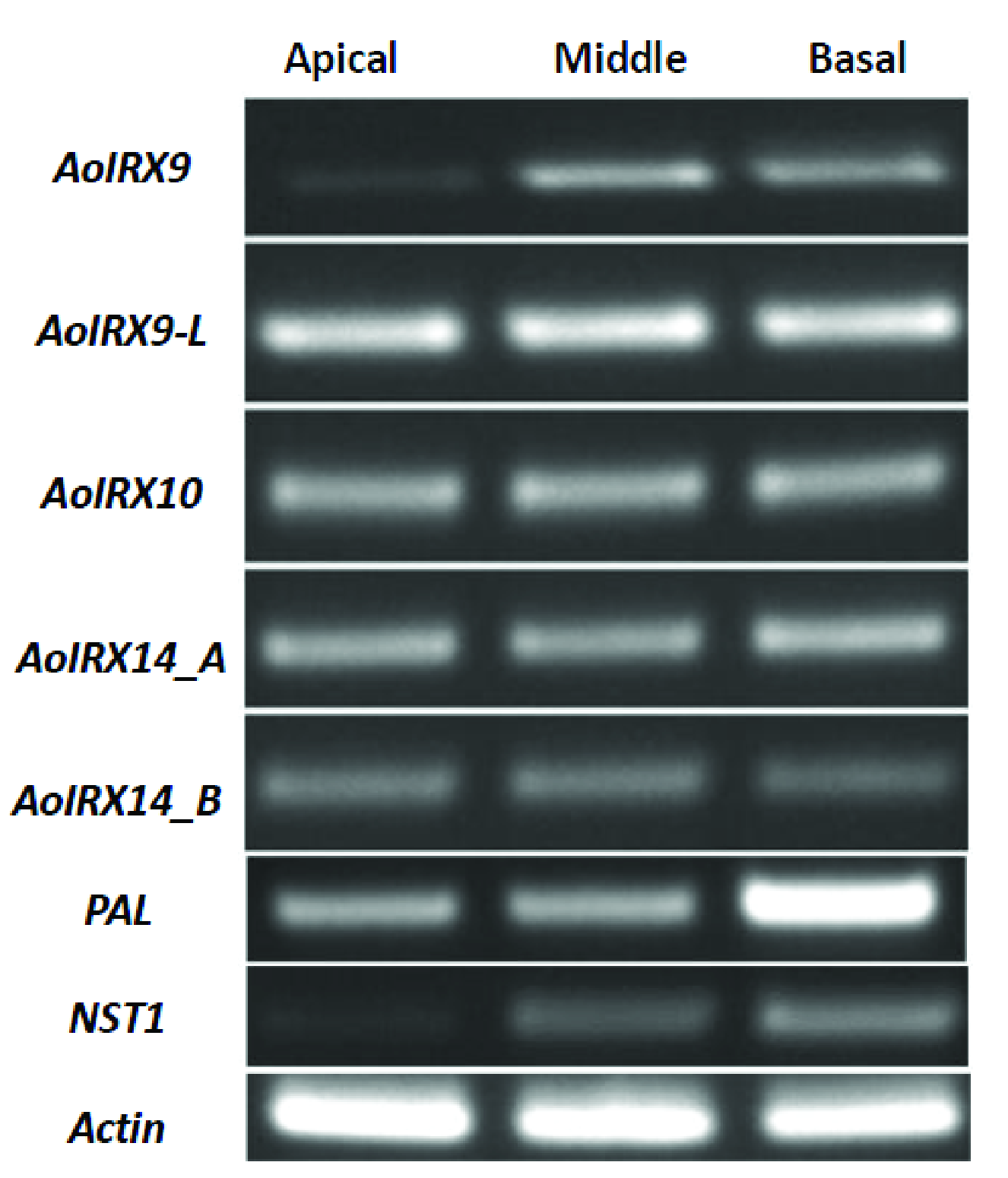

Supplement: S3 Fig — Total RNA was extracted from apical, middle and basal sections of fresh Asparagus spears and the expression patterns of AoIRX9, AoIRX9-L, AoIRX10, AoIRX14_A, AoIRX14_B, PAL and NST1, were analyzed by semi-quantitative RT-PCR using actin as a control. (TIF) [file pone.0123878.s003.tif]
