# Supplementary material for: Asparagus Spears as a Model to Study Heteroxylan Biosynthesis during Secondary Wall Development
Source: PLoS One. 2015 Apr 20;10(4):e0123878. doi: 10.1371/journal.pone.0123878 (PMC4404143; doi:10.1371/journal.pone.0123878)
Supplement: S1 Table — (DOC) [file pone.0123878.s004.doc]

**S1 Table: Monosaccharide linkage analysis of AIR from *Asparagus* basal (B), middle (M) and apical (A) spears**

| **Polysaccharide Calculations** | | | | | **Fresh** | | | **Stored** | | |
| --- | --- | --- | --- | --- | --- | --- | --- | --- | --- | --- |
|  |  |  |  |  | **B** | **M** | **A** | **B** | **M** | **A** |
| **Pectic Polysaccharides** | | | | |  |  |  |  |  |  |
|  | **Arabinan** | | 1,5-Ara (f) | | 3.6 | 11.0 | 22.7 | 2.8 | 8.0 | 14.8 |
|  |  | | 1,2,5-Ara (f) | | 0.0 | 1.2 | 0.0 | 0.7 | 1.5 | 1.8 |
|  |  | | t-Ara | | 0 | 1.2 | 0.0 | 0.7 | 1.5 | 1.8 |
|  |  | | **Total Arabinan** | | **3.6** | **13.4** | **22.7** | **4.2** | **11.0** | **18.4** |
|  | **Type I AG** | | 1,4-Gal (p) | | 4.6 | 10.2 | 10.3 | 2.1 | 3.1 | 3.8 |
|  |  | | **Total Type I AG** | | **4.6** | **10.2** | **10.3** | **2.1** | **3.1** | **3.8** |
|  | **Type II AG** | | 1,3-Gal (p) | | 0.0 | 0.0 | 0.0 | 0.0 | 0.0 | 0.0 |
|  |  | | 1,6-Gal (p) | | 0.0 | 0.0 | 0.0 | 0.0 | 0.0 | 0.0 |
|  |  | | 1,2-Ara (f) | | 1.1 | 1.4 | 1.4 | 0.9 | 1.6 | 2.2 |
|  |  | | 1,3,6-Gal (p) | | 0.7 | 0.7 | 0.7 | 0.5 | 1.0 | 1.2 |
|  |  | | t-Ara | | 0.7 | 0.7 | 0.7 | 0.5 | 1.0 | 1.2 |
|  |  | | **Total Type II AG** | | **2.5** | **2.8** | **2.8** | **1.9** | **3.6** | **4.6** |
| **Heteroxylan** | | | 1,4-Xyl (p) | | 44.0 | 12.0 | 2.8 | 46.3 | 16.5 | 5.4 |
|  |  | | 1,2,4-Xyl (p) | | 2.2 | 0.9 | 1.8 | 1.6 | 1.2 | 0.6 |
|  |  | | 1,3,4-Xyl (p) | | 0.7 | 0.3 | 0.8 | 0.6 | 0.3 | 1.2 |
|  |  | | 1,2,3,4-Xyl (p) | | 3.2 | 4.3 | 2.9 | 1.9 | 7.3 | 5.0 |
|  |  | | t-Ara* | | 4.6 | 5.9 | 8.4 | 3.5 | 6.3 | 6.3 |
|  |  | | **Total Heteroxylan** | | **54.7** | **23.4** | **16.7** | **53.9** | **31.6** | **18.5** |
| **Heteromannan** | | | | 1,4-Man (p) | 3.0 | 6.3 | 5.7 | 3.8 | 7.6 | 7.5 |
|  |  | | 1,4-Glc (p) | | 3.0 | 6.3 | 5.7 | 3.8 | 7.6 | 7.5 |
|  |  | | **Total Heteromannan** | | **6.1** | **12.6** | **11.5** | **7.6** | **15.2** | **15.0** |
| **Xyloglucan** | | | 1,4,6-Glc (p) | | 1.1 | 1.8 | 1.5 | 0.7 | 1.3 | 2.5 |
|  |  | | 1,4-Glc (p) | | 1.1 | 1.8 | 1.5 | 0.7 | 1.3 | 2.5 |
|  |  | | 1,2-Xyl (p) | | 1.1 | 1.8 | 1.5 | 0.7 | 1.3 | 2.5 |
|  |  | | t-Gal | | 1.1 | 1.8 | 1.5 | 0.7 | 1.3 | 0.9 |
|  |  | | **Total Xyloglucan** | | **4.8** | **7.2** | **6** | **2.8** | **5.2** | **8.4** |
| **Cellulose** | |  | 1,4-Glc | | 12.1 | 19.6 | 13.5 | 18.5 | 17.0 | 18.4 |
|  |  | | **Total Cellulose** | | **12.1** | **19.6** | **13.5** | **18.5** | **17.0** | **18.4** |
|  |  | | **Total** | | **88.4** | **89.2** | **83.5** | **91.0** | **86.7** | **87.1** |
| **Unassigned linkages** | | | | | | | | | | |
| t-Ara (f) | | | | | 0.0 | 0.0 | 2.4 | 0.0 | 0.0 | 0.0 |
| t-Xyl (p) | | | | | 5.3 | 7.8 | 9.5 | 4.2 | 9.1 | 10.7 |
| t-Glc (p) | | | | | 0.9 | 0.9 | 1.2 | 0.7 | 1.2 | 2.0 |
| t-Gal (p) | | | | | 0.4 | 0.6 | 1.4 | 0.4 | 0.3 | 0 |
| 1,2-Xyl (p) | | | | | 5.0 | 1.2 | 1.9 | 3.2 | 2.8 | 0.6 |
| **Other calculations** | | | | | | | | | | |
| unsubstituted xylan  =1,4-Xyl | | | | | 44.0 | 12.0 | 2.8 | 46.3 | 16.5 | 5.4 |
| substituted xylan  =2,4-Xyl+3,4-Xyl+2,3,4-Xyl | | | | | 6.2 | 5.5 | 5.5 | 4.1 | 8.8 | 6.7 |
| % Substituted xylan  =(2,4-Xyl+3,4-Xyl+2,3,4-Xyl)/(1,4-Xyl+2,4-Xyl+3,4-Xyl+2,3,4-Xyl)*100 | | | | | 12.3 | 31.4 | 66.3 | 8.1 | 34.7 | 55.4 |

* The balance of the terminal residues are likely to be t-GlcA that was measured colourimetrically to be approx. 10% (w/w) but were not detected in this neutral methylation.
