# Supplementary material for: Asparagus Spears as a Model to Study Heteroxylan Biosynthesis during Secondary Wall Development
Source: PLoS One. 2015 Apr 20;10(4):e0123878. doi: 10.1371/journal.pone.0123878 (PMC4404143; doi:10.1371/journal.pone.0123878)
Supplement: S2 Table — (DOC) [file pone.0123878.s005.doc]

**S2 Table: Real-time PCR primers of *Asparagus* xylan synthase and actin genes.**

| **Gene** | **Forward** | **Reverse** |
| --- | --- | --- |
| *AoIRX9* | 5’ TACCAAGGACCCTGACTGCT 3’ | 5’ GGGGTGATGACTAATGTTCCA 3’ |
| *AoIRX9-L* | 5’ GCCGATCTTCTTCCTCTGTTT 3’ | 5’ TGCCTCCATATGTGAATTTGC 3’ |
| *AoIRX14_A* | 5’ CAAATGGGAAAGCTGGCTAC 3’ | 5’ CACCCGTAACCAAAACCAAC 3’ |
| *AoIRX14_B* | 5’ AACAAATTGCCAGGGACAAG 3’ | 5’ GTAGCCCAAGTGACCACCTC 3’ |
| *AoIRX10* | 5’ TTTAAAGGTGCAATTTGGATG 3’ | 5’ TTCGAATCCCCAACATAAACA 3’ |
| *PAL* | 5’ TTCGACAAGGTGTTCAATGC 3’ | 5’ TTCGACAAGGTGTTCAATGC 3’ |
| *NST1* | 5’ GATGGGTTGTATGCCGAGTT 3’ | 5’ GGTGGTGGTGGTGATAAAGG 3’ |
| *Actin* | 5’ GGCTGGTATTTTGCCTTCAA 3’ | 5’ CCGCAACACAAAATACGTGA 3’ |
